# Variability and dilemmas in harm reduction for anabolic steroid users in the UK: a multi-area interview study

**DOI:** 10.1186/1477-7517-11-19

**Published:** 2014-07-02

**Authors:** Andreas Kimergård, Jim McVeigh

**Affiliations:** 1 Addictions Department, National Addiction Centre, Institute of Psychiatry, King's College London, 4 Windsor Walk, London SE5 8BB, UK; 2 Centre for Public Health, Liverpool John Moores University, Henry Cotton Campus, Level 2, 15-21 Webster Street, Liverpool L3 2ET, UK

**Keywords:** Harm reduction, Needle and syringe programmes, Anabolic steroids

## Abstract

**Background:**

The UK continues to experience a rise in the number of anabolic steroid-using clients attending harm reduction services such as needle and syringe programmes.

**Methods:**

The present study uses interviews conducted with harm reduction service providers as well as illicit users of anabolic steroids from different areas of England and Wales to explore harm reduction for this group of drug users, focussing on needle distribution policies and harm reduction interventions developed specifically for this population of drug users.

**Results:**

The article addresses the complexity of harm reduction service delivery, highlighting different models of needle distribution, such as peer-led distribution networks, as well as interventions available in steroid clinics, including liver function testing of anabolic steroid users. Aside from providing insights into the function of interventions available to steroid users, along with principles adopted by service providers, the study found significant tensions and dilemmas in policy implementation due to differing perspectives between service providers and service users relating to practices, risks and effective interventions.

**Conclusion:**

The overarching finding of the study was the tremendous variability across harm reduction delivery sites in terms of available measures and mode of operation. Further research into the effectiveness of different policies directed towards people who use anabolic steroids is critical to the development of harm reduction.

## Introduction

Needle and syringe programmes (NSPs) were introduced around the mid-1980s in response to the threat of HIV transmission amongst intravenous opioid injectors from the sharing of contaminated needles [1]. As a public health policy, the primary role of these programmes is to provide injecting drug users (IDUs) with access to sterile needles and syringes to reduce sharing and prevent transmission of blood-borne viruses. After more than two decades of research, evidence in support of this approach in preventing HIV and hepatitis C infections amongst psychoactive drug users is relatively robust [2-4].

Needle distribution can take place from fixed-sites services, such as drug agencies, community-based pharmacies, hospitals and vending machines, and mobile sites like outreach vans. Many NSPs combine needle distribution with other measures including health education, referral, screening for blood-borne viruses, counselling, opioid substitution treatment and safe disposal of used injecting equipment [2,5,6]. Broadly, these strategies fall under the definition of harm reduction, that is, interventions, programmes and policies aiming to minimise drug harms in individuals and society [2].

Although NSPs were initially established to serve injectors of psychoactive drugs, data from programmes in the USA [7], Australia [8] and the UK [9] show an increase in the number of anabolic steroid-using clients. While longitudinal data on steroid-using client attendance in NSPs is unavailable for many areas, in Merseyside, North West England, the number of new steroid-injecting clients increased sixfold from 1991 to 2001 [9], with this appearing to be an ongoing trend [10]. In many areas of the North of England, injectors of anabolic steroids and associated image and performance enhancing drugs (IPEDs) now constitute the majority of clients, with data from local authorities indicating that in some areas over 80% of clients report the use of IPEDs (Table 1).

**Table 1:** Percentage of NSP clients using IPEDs in the North of England-2014

**Area**	**Percentage**
Middlesbrough	67
Kirklees	60
Sheffield	62
Newcastle	52
Sunderland	60
Bradford	41
Halton	86
Liverpool	83
Sefton	43
St. Helens	34
Warrington	86
Wirral	77
Manchester	60
Bolton	52

Data provided by NSP service providers/managers via pied-forum@googlegroups.com. Presented by JM at the Public Health and Enhancement Drugs Conference, 9 April 2014, Liverpool.

Steroid users first accessed NSPs in some parts of the UK around the late 1980s [11]. Since then, NSPs for users of anabolic steroids and other IPEDs have become an established element of the service provision for drug users in the UK. Alongside conventional NSPs, other types of harm reduction interventions and programmes for steroid users have emerged such as outreach programmes in gyms where harm reduction service providers are handing out sterile needles and syringes to steroid users and facilitating access to other types of health care. The exact number of outreach programmes in the UK is unknown; however, a 2008 survey by the National Treatment Agency for Substance Misuse^a^ found that specific services for steroid users, including mobile needle distribution programmes in gyms and so-called steroid clinics, had been developed or were in the process of being developed in many parts of England [12]. Still, outreach programmes in gyms is not a new phenomenon with research suggesting that needle distribution programmes in gyms have been in operation since at least the early 1990s [11], such as a programme initiated in 1992 in South East England providing peer-led needle distribution to steroid users through a network of gym owners and dealers (AK, personal communication). For years, these informal distribution networks were widely used to reach anabolic steroid users, exceeding by far needle distribution to steroid users from fixed-sites NSPs. As a consequence, opportunities for service providers to engage directly with this user population were limited [6]. Steroid clinics are programmes designed for steroid users. These clinics typically provide sterile needles and syringes, information material about safe injecting and blood-borne viruses screening. Other interventions may include advice about weight training and diet to bring about desired improvement of physical appearance to avoid continuing drug usage. In some clinics, service providers offer medical examinations, like blood pressure monitoring and liver function tests, and advise steroid users to reduce dosage or not to use drugs at all in case of negative results [13]. Service delivery sites may offer interventions for opioid and stimulant users as well as steroid users in the same facility whereas some have opening hours reserved exclusively for steroid users. Other steroid clinics are set up in facilities separate from conventional NSPs such as the Drugs in Sport Clinic and Users' Support (DISCUS) which opened in 1994 [10].

While the use of IPEDs is by no means a new phenomenon, recent years have seen a marked increase in the availability and utilisation of these drugs. Developments in global communication systems (in particular, the growth of the Internet), cheap mass-produced pharmaceuticals and weak regulatory and enforcement practices in many parts of the world have combined to create a lucrative illicit supply of these drugs [14]. Diverse sections of the population are obtaining relatively cheap drugs in an attempt to increase their muscle mass, reduce fat, enhance intelligence or beauty or to improve their mood [15]. Research shows that many steroid users take these substances to enhance their physique as well as body satisfaction and that a majority of them are males [14]. By contrast, these drugs appear to have limited appeal to women partly because the main effect of these drugs is increased muscularity and partly due to adverse drug reactions including masculinisation. Most steroid users take two or more types of anabolic steroids at the same time, known as ‘stacking’, in 6- to 12-week periods or longer, referred to as the ‘on’ cycle, which is usually followed by a period of abstinence, called the ‘off’ cycle [16]. Anabolic steroids are taken both orally and by injection, but unlike many other injectors of illicit drugs, anabolic steroids must be injected intramuscularly, rather than intravenously. Long needles with a wide bore are needed to inject viscous steroids into large muscles such as the gluteus maximus (buttocks) or vastus lateralis (thigh). In addition to the possible adverse reactions of anabolic steroid use, including acne, gynaecomastia, liver damage, harms to the cardiovascular system and impact on mood and behaviour [17], sharing injecting equipment can result in the transmission of blood-borne viruses such as HIV, hepatitis B and hepatitis C. Studies with steroid users in Sydney [18] and North East England [19] reported that 5% and 2.1%, respectively, had shared injecting equipment. One study conducted in South Wales found that 20% of steroid users reported syringe sharing [20]. A recent study found that 8.9% of IPED users from NSPs in England and Wales had shared injecting equipment [21]. Of particular concern is the fact that 1.5% were HIV positive (similar to levels of HIV prevalence found amongst injectors of psychoactive drugs in the UK), 8.8% had been infected with hepatitis B and 5.5% with hepatitis C, highlighting that this particular group of IDUs is at risk of blood-borne virus infection.

The extent of harm reduction utilisation by anabolic steroid users is increasing, and the potential for harm in this group of drug users, including the transmission of blood-borne viruses, is in evidence. However, while a growing body of research has focussed on harm reduction for psychoactive drug users [6,22,23], studies into harm reduction policies for users of anabolic steroids, including outreach programmes and steroid clinics, are severely limited. This present study was conducted to explore existing interventions and the mode of operation in harm reduction services for anabolic steroid-using clients.

### Literature review: variability in harm reduction policies

Malins et al. [24] focussed on gender-specific risks in IDUs and found that women would try to avoid being placed in the marginalised category of a ‘junkie’, for example, by finding a secluded site when injecting. While this strategy enabled them to avoid police, other IDUs and the gaze of the general public, it also placed them at risk of not being found in case of an overdose. Similarly to gender-related risks reported by Malins et al. [24], it has been shown that body dissatisfaction, along with the desire to enhance muscularity, can trigger the use of anabolic steroids in males [16,25]. This exposes users to potential health harms, including from the use of multiple IPEDs [16], hazards relating to needle sharing [21], infections and injuries at injection sites [26], as well as blood-borne virus exposure associated with past injection of heroin and imprisonment [21,27]. In these ways, IDUs form subgroups with specific risk profiles, not just in relation to their drugs of choice but also their characteristics and surrounding environment, highlighting that social context is crucial to understanding drug-related harm [28]. These subgroups may, in turn, affect the evolution of harm reduction interventions and programmes. This is the case, for instance, with regards to the implementation of steroid clinics [13].

Issues relating to the stigmatisation of drug users can have wide implications for harm reduction. In a study of how stigmatisation influenced IDUs in a city in South West England, Simmonds and Coomber [29] found that groups of IDUs tended to stigmatise other groups of IDUs. For example, steroid users looked down at those they believed engaged in riskier behaviours than themselves. One potential outcome of this is that steroid users may ignore their own risky behaviours by comparing their practices with intravenous drug users who they perceive as engaging in far more dangerous activities [29]. The sense of stigma surrounding harm reduction service sites can also be far-reaching. Smith [30] explored oppositional strategies in response to the opening of a methadone clinic in Toronto and found explicit forms of stigmatisation directed towards clients who accessed the facility. In this process, stigmatisation of IDUs extended from stigma associated with the methadone clinic perceived as a site of disease and disorder [30]. Given that steroid users do not generally identify themselves with other IDUs [31], over the years, it is likely that some have kept away from NSPs, and some continue to do so today, in order to avoid being labelled a ‘drug user’. In response, tailored harm reduction programmes emerged to overcome such barriers; for example, the Steroid Peer Education Project in Victoria, Australia, commenced with one bodybuilding peer worker who visited gyms to deliver sterile needles and syringes to steroid users who rarely came in contact with fixed-sites NSPs [32].

Attitude amongst harm reduction service providers towards the perceived benefits of NSPs and towards IDUs, along with their harm reduction knowledge, have significant implications for the operation of harm reduction programmes. Tsai et al. [33] surveyed service providers in Taiwan and found that a positive attitude towards the benefits of NSPs influenced service implementation. Service providers who offered needle return services, referral, health education and screening services had a higher attitude score in terms of the benefits of NSPs compared to those who did not provide these services. By considering individual characteristics of service providers, focus is directed at those in charge of policy implementation. As Lipsky [34] notes, ‘street-level bureaucrats’—public service providers like teachers, policemen, doctors and social workers—have significant influence on the execution of policies based on their attitudes, moral beliefs, knowledge, interpretation of policy and routines. This is evident in the work of Spittal et al. [22], exploring needle distribution practice in Vancouver. Here, it was found that service providers used an informal ‘loaner system’ of injecting equipment when IDUs did not have any needles to return and hence were not allowed to obtain new injecting equipment. In contrast to such restrictive needle and syringe distribution policy—one new needle exchanged for one used that is returned—in other cases, service providers will not require that used needles are returned before providing sterile ones [35]. Clearly, there are many legitimate reasons why IDUs do not always return used injecting equipment, such as having disposed of their used needles and syringes in other services (including at hospitals). Regardless of the difference between local practice, these examples serve to highlight that harm reduction polices are carried out through relationships between service providers and clients where decision-making processes employed by service providers have tremendous influence on policy [34].

This article extends these insights regarding harm reduction service delivery to suggest that multiple factors, including the risk profile and behaviour of clients, social context of harm reduction and drug use, along with the decision-making of service providers, affect service implementation. As a result, the organisation of harm reduction programmes may vary significantly across delivery sites, for example, in relation to number of hours open, fixed-sites or mobile service delivery, number of clients, client demographics, needle and syringe distribution policies, range of interventions and availability of specific interventions for IDU subgroups such as young injectors, sex workers, homeless or IPED users [5,6]. An important issue here is that a growing number of studies indicate that impact from availability of interventions and operational procedures on the effectiveness of harm reduction can be wide-ranging [22,36-38]. An equally important issue, particularly in relation to this study, is that individual, social and institutional processes make harm reduction policy in practice highly complex. Hence, dilemmas, tensions and barriers in policy execution may likely arise.

## Methods

This article is based upon 6 months of research in 2009 and 2 months in 2011 as part of a study of drug policy aimed towards the illicit use of anabolic steroids. The primary method of data collection used qualitative interviews with service providers as well as steroid users (conducted by AK). The study was designed to explore harm reduction for steroid users in sites classified as (i) conventional NSPs set up chiefly for intravenous drug users, (ii) outreach programmes for steroid users and (iii) steroid clinics. Given the differences from area to area in terms of availability of harm reduction interventions and policies, the study included respondents from different parts of England and Wales (Table 2).

**Table 2:** Overview of data collection

**Site**	**Harm reduction programme**	**Interview with steroid users (yes/no)**	**Interview with service providers (yes/no)**	**Area**
1	Steroid clinic	Yes	Yes	North West England
2	Steroid clinic	Yes	Yes	London
3	Steroid clinic	Yes	Yes	Removed due to issues of confidentiality
4	Conventional NSP	Yes	No	North West England
5	Outreach programme for steroid users	Yes	No	Removed due to issues of confidentiality
6	Conventional NSPs^a^	Yes	No	South Wales
7	Conventional NSP	No	Yes	North West England
8	Steroid clinic	No	Yes	North West England
9	Conventional NSPs^a^	No	Yes	North Wales

^a^NSPs presented together to protect anonymity.

Interviews were conducted with nine service providers who had personally taken part in service provision for anabolic steroid users. From the review of the literature into the various aspects of harm reduction service provision, three overall interview topics were developed: (i) training of harm reduction service providers relevant to steroid use, (ii) health risks amongst steroid users—personal and anecdotal accounts and (iii) new and existing interventions specifically aimed at steroid users. Although these overall topics guided the interview questions, interviews were semi-structured; for example, all respondents were asked what harm reduction meant to them allowing for new perspectives on the content of this policy to be brought up. In this article, emphasis is on the findings from interviews with service providers.

Twenty-four anabolic steroid users were interviewed. Most of them used multiple steroids with nine reporting use of synthetic growth hormones. Interviews were semi-structured exploring attitudes, knowledge and experiences of steroid users while covering the following topics: (i) body (dis)satisfaction; (ii) motivations for use; (iii) patterns of steroid use, sources of steroid information and health harms and (iv) previous and current experience with harm reduction programmes. These broad topics were based on a review of literature reporting the experiences, viewpoints and practices of this group of drug users. This present article focusses predominantly on findings relating to category (iv), whereas other findings are reported elsewhere [39].

### Recruitment

Service providers were contacted by e-mail or telephone and asked to take part in the study. Sampling was done to ensure that service providers from different types of harm reduction programmes were included. As the study developed, service providers told of other delivery sites and through this ‘snowball’ sampling technique further respondents were included. Nine steroid users were recruited from steroid clinics, seven from NSPs and three from an outreach programme. One was recruited at a gym and four steroid users were recruited from a prison. In steroid clinics and NSPs, service providers approached clients and asked if they would be willing to partake. Very few declined. These interviews typically took place in a private room at the facilities. In addition, outreach workers had asked clients beforehand if they would take part.^b^

### Sample characteristics

The sample of service providers (four males and five females) included five agency-trained respondents, two nurses and two doctors. Respondents were affiliated with drug agencies and NHS services, respectively. Service providers were relatively experienced as many had been involved in harm reduction for more than 10 years. However, knowledge about and experience with harm reduction for steroid users varied significantly. Six respondents were involved in steroid clinics whereas three worked in conventional NSPs. Most of their work involved service provision for other IDUs.

The mean age of steroid users was 34 years. As noted, most steroid users are male, which is reflected in the sample that consisted of 24 males. The sample of steroid users included those on their first visit as well as individuals who had been accessing services for more than 10 years. Twelve of them were currently or had previously attended conventional NSPs, while three made use of an outreach programme and nine of steroid clinics. The majority of the samples were working, with occupations including doorman, taxi driver, gym owner, self-employed business person, construction worker, IT-project manager, fitness instructor, crane operator and university student.

### Analysis

Interviews with service providers were transcribed in full. Interviews with steroid users were transcribed as well, except when prison rules prohibited electronic devices and when respondents felt uncomfortable about being recorded when discussing sensitive issues of anabolic steroid use. In such cases, a written recording was made immediately after the interview was finished. All written interview records were subjected to thematic content analysis in order to identify and verify themes relevant to the operation of harm reduction services allowing for the framework of analysis to be adapted as the study progressed [40].^c^ During the analysis, comments relating to needle distribution, health education, harm reduction measures and customised harm reduction policies were categorised as the study evolved (Tables 3 and 4). The analysis was led by AK with input from JM to compare and contrast the results.

**Table 3:** Coding framework (selected examples): from interview records to initial coding

**Interview transcript**	**Initial coding**
Interviewer: So who injects you?	
Steroid user: My mate does it. I got him to come here [in the service] with me. So I got him started on doing it, so we both came here and got them [service providers] to show how he should do it.	Users' injecting practices Available interventions
	Conflicts in perspectives and practices (service providers recommended that users inject themselves)
Interviewer: When you go to the service, you're just asking for syringes and needles?	
Steroid user: I don't think they know that much about the steroids in those sort of places. You could put a bit more information about certain things in them leaflets. I think they're limited in what they actually will tell you. It's all negative.	Users' viewpoints on harm reduction services
	Negative viewpoints about conventional needle and syringe programmes
Interviewer: Where does your sense of responsibility [towards clients] come from?	
Service provider: Because people associate it [steroid use] with health, because people are going to the gyms and they work out. So people think of it as being healthy, well it's not. It's like it's okay, because you are not injecting into a vein and costing us a lot of money.	Harms relating to steroid use
	Perceived differences between steroid users and opioid users
	Perceived need for services
Interviewer: What extra services do you provide?	
Service provider: We do syringe exchange, safer injecting information, the nurse will give them hepatitis B vaccinations, complete blood count [to determine infection], liver function tests, cholesterol tests, dietary advice, blood pressure monitoring and safer drug use messages. Even smoking cessation, the nurse will look at that as well. We do, recently, chlamydia and gonorrhoea screening.	Interventions in steroid clinics
	Scope of harm reduction
Interviewer: If you should sum up harm reduction, what is the definition you would give?	
Service provider: What the tendency is: ‘What was the mistake I made? I must not have taken enough [amount of anabolic steroids], so next time I'll do it. I'll increase the dosage’, and they go through the same process, get more side effects. We try to educate people about the whole thing, the whole package, so that they are able to maximise the effects. If they are going to do it, then they should get the result [in physique] and maintain those results. The idea being that if they are happy with the results they get, they are less likely to then go on to higher doses or more cycles.	Dosage information
	Offer information/interventions to limit usage
	Development of specialised interventions
	Notions of steroid clinics

**Table 4:** Coding framework (selected examples): from reduced categories to final coding framework

**Refined and reduced categories**	**Final coding framework**
Injection injuries	Perceptions of harm (in steroid users)
Adverse effects	
Notions of harm in intravenous/psychoactive drug users	
Advice from service providers	Disparity between using practices (service providers contra steroid users)
Preferences and viewpoints amongst users	
Negative peer influence	
Perceptions of syringe distribution limitation	Needle and syringe distribution models
Experiences with outreach-based distribution through users	
Notions of key users as distributors	Boundaries of harm reduction
Steroid regimen information	
Positive outcome of steroid regimen information	
Negative views on steroid regimen advice	Steroid clinic function
Steroid clinics versus conventional needle and syringe programmes	
Positive notions of specialised interventions	
Negative outcome of specialised interventions	

The study was approved by Liverpool John Moores University Research Ethics Committee. Respondents were given a brief outline of the study and guaranteed anonymity before being presented with a consent form.

## Results

In talking to harm reduction service providers, it became clear that they all had experienced an increase in the number of steroid users in their local area:

…should we give out needles to steroid users? And I think: yes, we should. Because there is many people using it. It's probably the number one drug of choice right now. (Female service provider, steroid clinic, site 1)

In addition, they all reported various risk behaviours of steroid users such as inadequate injection technique and needle sharing—although service providers' reports of sharing varied significantly:

They learn to inject from one another. So if you got someone who hasn't developed a good technique then that will be passed on as well…sometimes they will be using too short a needle and not leaving enough to stick out in case it snaps off…The whole sharing of equipment, we know that is going on. We still hear these tales about where the gym owner has pre-filled syringes under the counter and walks out and injects gym members. (Female service provider, NSP, site 9)

Aside from these two main findings, four themes emerged from the analysis of the accounts of service providers and steroid users (dealt with below):

(i) All steroid users reported having easy access to sterile needles and syringes from harm reduction service sites. However, when steroid users were asked about their injecting practices, they tended to ignore or at least make their risky behaviours seem less hazardous than they actually were, impeding, in some cases, the uptake of advice about safe injections.

(ii) Secondary distribution involves distribution of sterile needles and syringes through social networks of IDUs and has been shown to improve needle coverage in hard-to-reach groups of opioid users [6,41]—although care must be taken when extrapolating this finding to steroid users. On the one side, secondary distribution was attributed with greater access to sterile injecting equipment in hard-to-reach groups of steroid users and on the other side service providers expressed concern because this approach limited engagement with vulnerable groups of young steroid users. It became obvious that service providers faced a trade-off between high needle availability and personal contact with users.

(iii) When talking to service providers, disagreements about the boundaries of harm reduction for steroid injectors arose. A few service providers provided steroid users with information about the use of specific types of anabolic steroids whereas a majority rejected this policy. The dilemma here is that service providers may offer this information in the hope that steroid users will come to them for advice or they may choose not to and thereby leaving users with potentially uncertain information from peer users in gyms.

(iv) Availability of interventions designed particularly for steroid users varied across services. However, in steroid clinics, the use of medical examinations of steroid users was well established. The aim of medical examinations was to make users aware of adverse reactions to their use of anabolic steroids. Yet it was clear that users who underwent periodic examinations also felt less at risk from the use of these drugs.

### ‘We offer advice around injection technique’

It was clear that steroid users in this study saw themselves as different from other IDUs. In their opinion, steroid users do not share used needles with other users whereas this was believed to be the case with users of opioids and stimulants:

No bodybuilders that I know share. I mean you hear of what are called druggies, people who use other stuff. I don't mix in those circles to be honest. (Steroid user, NSP, site 6)

It came across that the drugs steroid users were taking seemed to create these differences. One steroid user expressed that ‘steroids aren't addictive, it's a hormone’ (Steroid user, NSP, site 6). From his perspective, steroid users would not therefore use these drugs compulsively and would not therefore borrow needles from other users—even though weight training, motivations to improve body image satisfaction and IPED usage appear to be characterised by obsessive and compulsive behaviour in some anabolic steroid users [42]. In these ways, it seemed that steroid users attempted to distance themselves from risk behaviours practiced by intravenous drug users, similarly to what Simmonds and Coomber [29] found in their study of needle distribution policies and IDUs in South West England. Although steroid users tended to view the potential hazards of steroid use as minimal, this did not mean that they did not practice risky behaviour. For instance, two steroid users admitted to having reused their injecting equipment. Several had suffered ‘lumps’ or abscesses at the injection site which may have been caused by incorrect injecting technique or from the use of non-sterile products. Furthermore, a service provider noted complications of injecting into the buttock:

I asked if his [client's] injecting technique was okay and he said: ‘Yes, I’ve been injecting for a number of years.’ I said: ‘Just humour me and show me where you have been injecting’. So he said: ‘Right in the middle of my buttock.’ I said: ‘Well you do realise the sciatic nerve runs very close to where you are putting your finger? He said: ‘Oh, yes. I think I might actually have hit it a couple of times.’ (Male service provider, steroid clinic, site 2)

Other service providers expressed concern with the use of small needles that might break inside the muscle:

That needle is too bendy to go into a muscle and it's designed for intravenous use. So I rather advise them not to use it in case it ‘snaps’. (Female service provider, steroid clinic, site 1)

However, while service providers in this study generally encouraged clients to use a large needle for steroid injections, steroid users had many reasons for why they preferred to use a smaller one instead, such as being worried that a large one would cause them pain: ‘there is no way I'm putting that [needle] up my arse’ (Steroid user, steroid clinic, site 1). In other cases, the decision to use small needles was based on advice on injecting technique from other users.

These last few accounts provide a sense of self-assurance amongst steroid users regarding their abilities to conduct safe injections. The important issue here is that self-assurance in steroid users appeared to displace acknowledgement of their risky behaviour—a finding which shares some similarities with those of other studies indicating that the harms of steroids are often trivialised or ignored by steroid users [43]. In addition, this also appeared to act as a barrier to the dissemination of safer injecting advice. In situations of disagreements between service providers and steroid users, service providers tended to fulfil the requirements of steroid users by handing out small needles to maintain rapport with steroid users and ensure access to sterile injecting equipment.

### ‘Needle exchange is a core provision for us’

Most service providers reported no restrictions on the number of syringes that could be provided per visit free of charge, reflecting an emphasis on high utilisation of sterile needles and syringes to prevent disease from being spread. Alongside traditional needle distribution, interviews showed that secondary distribution of needles and syringes was accepted practice to improve the use of sterile injecting equipment. For instance, in a number of harm reduction service sites, established steroid users, such as competing bodybuilders and gym owners, were allowed to collect large amounts of injecting equipment for themselves and for others. Furthermore, interviews with steroid users/gym owners gave insights regarding outreach-based needle distribution revealing that outreach workers provided them needles and syringes which were distributed to steroid users in these gyms. In some cases, outreach work had the added advantage of acting as a bridge to other harm reduction interventions as service providers would persuade users to visit conventional NSPs. In one NSP, peer-based needle distribution was based upon informal agreements between service providers and a client. Here, service providers provided a local steroid user and gym owner with needles and syringes. In turn, he had set up a ‘syringe exchange’ in his gym including placing sharp bins in the locker room. He would state his reasons:

…because most of the members are too embarrassed to go to the exchange [NSP] themselves, and I was tired of seeing used syringes in the locker room. (Steroid user, NSP, site 4)

These various models of needle distribution were clearly ways for service providers to improve sterile needle usage amongst IPED injectors through customised policies. What these policies share is that in the view of service providers, the credibility of key steroid users in the gym using population (gym owners/competing bodybuilders) enabled them to engage with steroid users who did not access conventional NSPs. This resonates with the positive experiences from the Steroid Peer Education Project, an outreach-based needle and syringe distribution service led by a bodybuilding peer worker [32].

However, in other harm reduction service sites, service providers were limiting the number of syringes per visit.^d^ Below, a service provider reflected on restrictions on needle distribution for young and inexperienced steroid users due to concern of the loss of opportunity for health education:

If you are giving a hundred needles and syringes to a young, uneducated steroid user, and if they are taking them to give out to other similar people, you are losing the opportunity to get involved with them. So you have to work with them slightly different to the older steroid user group who can be a bit more responsible in terms of their injecting. (Female service provider, Steroid clinic, site 8)

Constrained by limited funding, service providers in one NSP had started to ask for an economic contribution from steroid users to offset costs.^e^ While this financial contribution was voluntary, and users would supposedly be provided regardless, it was meant to offset costs of steroid users collecting large amounts of needles and syringes for themselves and for secondary distribution. Whereas this might be seen as an obstacle to widespread needle availability amongst some groups of IDUs this did not seem to be the case for this user, reflecting that most steroid users in this study were employed and were able to pay for a gym membership, food supplements and drugs:

…steroid users see it as great that they don't have to pay because that is more money for protein [food supplement]. But I must be honest, when she [service provider] told about a [financial] contribution [to offset the cost], I haven't got a problem with that, because otherwise we'd be forced to buy all these things across the Internet. (Steroid user, NSP, site 6)

It was clear that these different needle distribution policies were the result of local arrangements in service delivery sites. Many of them appeared to have been developed on an ad hoc basis which resonates with findings of Spittal et al. [22] on the evolvement of an informal ‘loaner system’ from the formal ‘one-for-one’ approach (one clean needle for one used returned). It is also in keeping with Lipsky's [34] views on the wide manoeuvre room of service providers in their interpretation of policy.

### ‘Give information about steroids' effects’

A substantial theme was where the line should be drawn in terms of which types of harm reduction interventions should be provided. As shown below, these different perspectives on the boundaries of harm reduction were widely dependent on service providers' knowledge of issues relating to anabolic steroids.

Syringe distribution, along with advice on safer injecting practices, was viewed as essential, with a number of service providers providing additional information about the potential harms of anabolic steroids—although it was apparent that service providers in steroid clinics knew considerably more about steroid-related harm than those in conventional NSPs. In five harm reduction delivery sites, steroid users were provided with dietary services and advice about weight training as service providers believed that this could help bring about the desired effect on steroid users' physique. This approach was based upon a view that at best, improvement of body satisfaction would persuade clients not to use steroids at all:

…quite a lot of the time people will take onboard that what they are doing in the gym is not correct, or their diet, and that they can actually make some really positive changes without even going on steroids. So what we tend to say is, ‘Okay, you are thinking about using them, but your diets and training aren't correct. Go away, change all of those and come back to see me in six months time and if you are not happy with the way you have progressed let us look at it again.’ (Male service provider, steroid clinic, site 2)

However, the effectiveness of this strategy in prolonging the time before the use of steroids is difficult to determine, as it is possible that some people will turn instead to established steroid users for advice. In this study, as with others [43], peer influence was high with steroid users relying heavily upon information about these drugs from each other, rather than from harm reduction service providers:

You go to the gym, you get involved with people, they give you advice, you can tell them what your own feelings are. I've never had any information off drug services, all my information has been from the guy I bought the steroids from. (Steroid user, NSP, site 6)

…the drug service is just a handy place to get your needles, literally that is all. (Steroid user, NSP, site 6)

In response to peer-driven information networks in gyms, a service provider in one steroid clinic argued that clients should be advised about the use of anabolic steroids as an alternative to the ‘hokum and misinformation’ from other steroid users (Male service provider, steroid clinic, site 2). In this clinic, steroid users were provided sheets containing information about specific types of anabolic steroids including dosage, pharmacological effects, ‘stacking’, period of usage as well as potential side effects. For this service provider, this information was seen as an essential component of harm reduction:

It's a harm reduction message that you need to be making people aware that if they take nandrolone [decanoate] on its own they are not going to have a sex drive for the next twelve weeks. If someone is using a particular toxic oral steroid you might wanna make them aware that if they combined it with another steroid they can actually reduce the dosage of the more toxic one…if you got a steroid that readily converts to oestrogen, for example Dianabol [methandienone], then you might be saying to them: ‘You need to very careful about gynaecomastia’, because it's a real potential issue for you with that one. (Male service provider, steroid clinic, site 2)

Again, interviews with service providers revealed conflicting perspectives on this policy. In fact, information about anabolic steroids was limited to one steroid clinic whereas a majority of service providers did not provide such advice. A concern amongst these service providers was the unknown effects from long-term use of high doses of anabolic steroids:

We wouldn't give them specific advice on the drugs they are taking, how to take them, when to take them all that sort of stuff because it's such an unknown quantity and generally they have that guidance from other users. Whether it is accurate or not I don't know but they seem to know more than we do. Our advice will be specifically health related. (Female service provider, NSP, site 9)

Arguably, the uncontrollable nature of the illicit market makes it difficult to know exactly the active substance in drugs obtained from the illicit market which is reducing the relevance of information about specific types of steroids [44].

### ‘This steroid clinic to me is vital’

Service providers explained that steroid clinics had been set up as a result of an increase in the number of steroid users accessing conventional NSPs. In addition, providing efficient harm reduction in this group of clients required tailored health interventions as depicted in this account of a service provider who is reflecting on the opening of a steroid clinic around the mid-2000s:

There were all these steroid users coming in, and they were just getting needles, and we were telling them how to inject safely…that was when we started talking, ‘Look, these people need to have their own clinic and special services and come in and get [medically] checked out.’ So that's why we brought it up. Then we just took it to our line-manager, who took it further up [in the organisation], and we got the okay. (Female service provider, steroid clinic, site 1)

It seemed that in many cases steroid clinics were created with input from anabolic steroid users. In one clinic, for instance, service providers conducted an informal survey amongst steroid using doormen as well as members of the local gym to determine which day and at what time steroid users might prefer to come in. In the view of service providers, responding to requests of steroid users is essential in gaining engagement from this group of IDUs.

It became apparent that steroid users tended to view service providers in steroid clinics as being non-judgemental ‘because you can come here and speak openly’ (Steroid user, steroid clinic, site 3). One steroid injector preferred to drive a relatively long way to engage with a steroid clinic specifically oriented to his needs even though he was living next to a conventional NSP. Steroid users spoke of issues that were interpreted as stigma in relation to NSPs set up to deal with psychoactive drug users which may explain why the steroid users in this study preferred steroid clinics. As seen below, the sense of stigma when accessing NSPs was seen by service providers as a particular problem in outlying areas because of issues of privacy which may further account for why users in remote areas prefer to travel to steroid clinics. These findings are in line with Smith's [30] discussion of the socio-spatial stigmatisation relating to drug treatment facilities.

If you live in a little village somewhere, and that is your only needle exchange, and you have family that lives there, works there, the whole thing about confidentiality goes out the window. Providing services in a remote area isn't the same as in big cities where loads of people are coming and going. (Female service provider, NSP, site 9)

One steroid clinic was open one afternoon per week in the same building as the conventional NSP. However, during this time, only steroid and IPED users were allowed to come in. This programme provided a variety of measures:

We do syringe exchange, safer injecting information, the nurse will give them hepatitis B vaccinations, complete blood count [to determine infection], liver function tests, cholesterol tests, dietary advice, blood pressure and safer drug use messages. Even smoking cessation, the nurse will look at that as well. We do, recently, chlamydia and gonorrhoea screening. (Female service provider, steroid clinic, site 1)

Service providers in steroid clinics explained that the aim of medical examinations of steroid users was to advise against high doses in case of adverse tests results. For example, if medical tests indicated high blood pressure, high levels of cholesterol or injury to the liver, steroid users could be advised to reduce dosage or not to use anabolic steroids at all until tests returned to normal. However, statements from a number of steroid users suggest that having access to medical examinations made them feel safer:

Obviously I get regular tests so if there is a problem then I know what actions to take to counter those problems. Where people who take steroids continually, and don't get their blood checked, obviously they are going to run some kind of health risk because anabolic steroids tend to be associated with problems with cholesterol and it is not a good idea to stay on them constantly. (Steroid user, steroid clinic, site 3)

According to this steroid user, actions to ameliorate potential health problems included the substitution of certain anabolic steroids with others—as different anabolic steroids have different pharmacological effects in the liver—and using cholesterol-lowering drugs. While medical examinations may lead to a positive change in drug-related behaviour amongst steroid users, including dose reduction, there is also a risk that these tests induce risky behaviour such as the use of multiple drugs including auxiliary drugs for the self-treatment of adverse steroid reactions.

## Discussion

This article reports on the function of harm reduction programmes for anabolic steroid users. The overarching finding was the tremendous variability in the delivery of harm reduction across service sites in terms of availability and execution of harm reduction measures. This was the result of multiple factors including service demand of steroid-using clients, the social context of harm reduction, as well as service providers' interpretations of harm reduction for steroid users. The variability amongst harm reduction programmes is in contrast to other areas of health service delivery, highlighting the absence of a national ‘best practice’ for harm reduction policies towards users of anabolic steroids. Another key finding was that variability amongst services, offering steroid users a greater choice of interventions in some sites, appeared to improve service uptake in these sites. In this way, the combination of a wide range of measures would seem to increase the ability of services to reduce harm in users, which is broadly consistent with findings from studies of harm reduction for psychoactive drug users [2,6]. An equally important finding of this study was significant dilemmas in the way that harm reduction service models are currently implemented that could be impeding the effectiveness of the existing interventions and policies.

The provision of sterile needles and syringes was seen as playing a pivotal role in the delivery of efficient harm reduction for steroid users. However, given the variety in models of needle distribution found in this study, it is possible that the coverage is greater in some areas than others; for example, whereas many service providers endorsed secondary distribution including peer-led arrangements with gym owners/competing bodybuilders to increase availability of sterile injecting equipment amongst steroid and IPED users, other service providers argued that needle distribution without contact is a missed opportunity for health education. Here, studies of needle distribution policies directed towards intravenous drug users suggest that the access to and utilisation of sterile injecting equipment increases in cases where several methods are being used to distributed needles and syringes [6]. In addition, studies indicate that widespread needle distribution is more effective in reducing blood-borne virus infections than restrictive syringe policies [37,38], without increasing unsafe disposal of contaminated needles [45]. However, given that peer-to-peer information exchange is high in groups of anabolic steroid users [43], users who rely solely on advice from other users will most likely learn risky behaviours. In terms of NSP evaluation, secondary distribution obviously makes it difficult to determine the number of sterile needles being provided by secondary distributors, used by the steroid-using population and disposed of safely. These are issues that will have to be addressed in future research.

The effects of stigma on IDUs appear sufficiently far-reaching to impede service uptake amongst steroid users. They do not wish to interact with or be connected to opioid and stimulant injectors in conventional NSPs. As noted by Simmonds and Coomber [29], steroid users clearly felt different from intravenous drug users and did not want to be mistaken for a ‘junkie’. An important finding was that steroid users found the steroid clinic attractive because of service providers' knowledge about and non-judgemental attitude towards the use of anabolic steroids. On a more practical level, steroid clinics may be better suited to maintain regular contact with steroid users, given that they offer more interventions, whereas evidence suggests that steroid users in NSPs make few visits per year because they inject more infrequently than intravenous drug users [9].

Evidence from the harm reduction literature regarding psychoactive drug users suggests an enhanced impact of harm reduction when multiple interventions are delivered in combination [2]. For example, the impact of NSPs on the reduction of blood-borne virus infection may be greater when delivered in combination with other interventions [23]. Likewise, steroid clinics may possibly achieve greater results in terms of reducing steroid-related harm than conventional NSPs because they provide steroid users a wide variety of measures at the same time. Perhaps then, the idea of one or two formal steroid clinics per region, with input from physicians and nurses, is worth exploring at this time to improve accessibility to a wide range of harm reduction measures.

However, a caveat to the promotion of steroid clinics is that few scientific investigations have been undertaken to identify the impact of steroid clinics on client risk behaviour. Importantly, this study has shown that information and advice from medical examinations of steroid users may not result in anticipated behavioural changes. It was also clear that medical examinations, including screening for liver damage caused by the pharmacotoxicological actions of anabolic steroids, tended to instil a sense of safety in steroid users. However, post marketing reports of liver failure with an authorised medicinal product suggest that liver function tests were not likely to have prevented cases of liver failure despite periodic liver monitoring of patients [46]. It is a concern therefore that periodic liver function tests of steroid users are not likely to detect liver damage, especially since gaps exist in what is currently known about liver harms caused by steroid usage [14]; for example, the period between the initial use of steroids and the potential onset of liver damage is largely unknown, making it difficult to administer the test at the right time. This concern resonates with work of Winstock et al. [47] arguing convincingly that interventions, in their case, pill testing of ecstasy tablets, which are unlikely to identify risks can falsely imply safety to users. Research into the outcome of interventions designed purposively for steroid users is therefore greatly needed to feed into the ongoing process of scaling-up access to, and achieving adequate coverage of, services providing a ‘comprehensive package’ of harm reduction for steroid users.

A caution of the present study is that many conclusions are based on interviews with service providers and it may be that they altered their answers to highlight their perceived benefits of harm reduction while downplaying difficulties of service delivery. Service providers seemed surprised, even embarrassed, when clients disclosed local infection such as abscesses from using the wrong type of needles. This could mean that service providers tended to keep these incidents to themselves. Another limitation is that this study offers little insights into barriers to accessibility of harm reduction service sites in steroid users who choose not to access these sites since all steroid users in this study had been in contact with harm reduction programmes. This latter point raises an important issue by bringing to attention the group of steroid and IPED users who avoid contact with harm reduction programmes and other health services, highlighting a need to develop targeted public health tactics to reach this population. In relation to this, qualitative in-depth research into ‘hidden’ and, possibly, high-risk groups of steroid users would be a useful direction for future research in relation to understanding the risk behaviour of these groups of users.^f^

## Conclusions

Support for harm reduction from service providers and anabolic steroid users suggest that these programmes are well-positioned to deliver health interventions to this group of IDUs. However, harm reduction policies only work when they bring about change leading to an overall reduction of harm [2]. Given the complexity of harm reduction policy delivery, as seen above, there are bound to be problems and barriers impeding service effectiveness. Yet more can be done to improve harm reduction. Nearly all of what is currently known about the dynamic relationship between the function of different harm reduction strategies and their effectiveness is derived from studies including users of opioids and stimulants, not people who use anabolic steroids. Due to the increase in the number of IPED injecting clients in harm reduction services, research is urgently needed to determine with greater accuracy the outcomes of harm reduction for steroid and IPED users including the effectiveness of syringe policies, such as peer-led needle distribution, and harm reduction interventions designed specifically for anabolic steroid users.

## Endnotes

^a^A policy advocacy body in England (now incorporated into Public Health England).

^b^No financial incentives were offered to steroid users.

^c^The article only presents quotes from interviews that were recorded on audio.

^d^This practice is in fact against National Institute for Health and Care Excellence (NICE) guidance on the optimal provision of NSPs amongst injecting drug users.

^e^This too is against NICE guidance.

^f^Given that the number of steroid and IPED users in the UK is unknown it is not possible to quantify harm reduction service uptake. In contrast, qualitative studies can be of use to acquire a better understanding of steroid users who are not in contact with any type of health services.

## Abbreviations

NSPs: Needle and syringe programmes; IDUs: Injecting drug users; IPEDs: Image and performance enhancing drugs; DISCUS: Drugs in Sport Clinic and Users' Support.

## Competing interests

Authors declare that they have no competing interests.

## Authors’ contributions

AK conceptualised the study. AK conducted the interviews and supervised the analysis with input from JM. AK wrote the first draft of the manuscript with subsequent contribution of JM. Both authors have read and approved the final version of the manuscript.

## Authors’ information

AK is a Principal Research Fellow at the Addictions Department, King's College London, as well as a Visiting Lecturer at Centre for Public Health, Liverpool John Moores University. His research covers the use and misuse of over-the-counter medicines as well as the use of anabolic steroids and other drugs for human enhancement purposes, along with related interventions, strategies and policies used to promote public health. JM is the acting director of Centre for Public Health, Liverpool John Moores University. He has worked as a practitioner and researcher in the field of anabolic steroids and associated drugs for over 20 years.
